# Improving ORB-SLAM3 Accuracy in Dynamic Scenes with YOLO11 Segmentation

**DOI:** 10.3390/s26051487

**Published:** 2026-02-27

**Authors:** Renata Raffaine Villegas, Anselmo Rafael Cukla, Gabriel Alejandro Tarnowski, Guillermo Mudry, Sergio Omar Lapczuk, Ely Carneiro de Paiva, Daniel Fernando Tello Gamarra

**Affiliations:** 1 Faculdade de Engenharia Mecânica, Universidade Estadual de Campinas (UNICAMP), Rua Mendenleyv, 200, Cidade Universitária, Campinas, São Paulo 13083-860, Brazil; elypaiva@fem.unicamp.br; 2 Departamento de Processamento de Energia Elétrica, Universidade Federal de Santa Maria (UFSM), Av. Roraima nº 1000, Santa Maria 97105-900, Brazil; anselmo.cukla@ufsm.br (A.R.C.); daniel.gamarra@ufsm.br (D.F.T.G.); 3 Departamento de Ingenieria Mecatrónica, Facultad de Ingenieria, Oberá CP 3360, Argentina; gabriel.tarnowski@fio.unam.edu.ar (G.A.T.); guillermomudry@gmail.com (G.M.); sergio.lapczuk@fio.unam.edu.ar (S.O.L.)

**Keywords:** visual SLAM, YOLO, ORB-SLAM3, ROS2

## Abstract

Traditional Visual SLAM systems, like ORB-SLAM3, often lose accuracy in dynamic environments. This work presents YOLO11-ORB-SLAM3, an enhancement to ORB-SLAM3 for dynamic scenarios, which integrates a YOLO11-based instance segmentation module to detect and exclude dynamic features from the tracking process. The system is designed to work with stereo and RGB-D cameras, and its performance was evaluated on challenging dynamic sequences of the public TUM RGB-D dataset, and also through real-world experiments on a mobile robot using a stereo camera to highlight its robustness and viability for real robotic applications. Experimental results demonstrate that the proposed system outperforms the original ORB-SLAM3, reducing the error by 93% in the public TUM dataset while preserving computational efficiency.

## 1. Introduction

With the development of robotic systems, the autonomy of robots is increasing. However, localization in completely unknown environments remains a major challenge. This is the main concern of SLAM (Simultaneous Localization and Mapping), in which the robot must build, update, and localize itself within a map based on its observations [1]. Compared to other sensors, cameras provide richer environmental information in real time, which is essential for applications, such as autonomous vehicles. Nevertheless, visual odometry (VO) and feature-based SLAM are sensitive to external factors—such as adequate lighting, textured surfaces, and static scenes—all of which affect the reliability of mapping and localization [2].

ORB-SLAM3 [3] is a widely used feature-based Visual SLAM (V-SLAM) algorithm that estimates robot motion and constructs a 3D map of the scene in real time by matching features in consecutive frames. Its ability to reuse previously generated maps after tracking failures has made it popular. Nevertheless, the algorithm is still vulnerable to dynamic environments: moving objects introduce unstable features that degrade feature matching and lead to drift or tracking loss. These limitations lead to a growing interest in developing strategies, such as using deep-learning-based segmentation to improve the performance of ORB-SLAM3 in non-static scenarios.

You Only Look Once (YOLO), for instance, can help filter dynamic objects. First proposed in 2015 [4], the algorithm has been continuously improved since then. YOLO11 introduces architectural refinements to improve both accuracy and computational efficiency [5], which makes it suitable for real-time SLAM applications. Several studies have investigated the integration of object detection or segmentation with SLAM. In comparison with other YOLO versions, YOLO11 replaces the C2f block with the C3K2 (Cross Stage Partial Kernel) block, which improves the computational efficiency by employing two smaller convolutions instead of one large convolution. For this reason, this version was selected to address the dynamic object removal.

The instance segmentation model provides object-specific masks along with their associated confidence scores and class labels. By identifying object classes, potential dynamic elements can be selectively excluded from the tracking pipeline, thereby optimizing the overall algorithm performance. For example, the approach in [6], which combines YOLO11-based detection with ORB-SLAM2, achieved improvements in dynamic environments, but remains limited by the older SLAM version and lacks support for other sensor configurations such as stereo cameras. Other methods explore semantic information in SLAM without focusing specifically on dynamic-object removal [7], while more recent implementations using YOLO11 have demonstrated promising segmentation results but do not support stereo input and do not operate in real time or on real robotic platforms [8]. Thus, despite these advances, existing approaches typically lack one or more essential elements: compatibility with both RGB-D and stereo cameras, maintenance of real-time performance, validation on a real robot and code availability.

To address these gaps, this work introduces YOLO11-ORB-SLAM3, an enhanced version of ORB-SLAM3, which integrates a YOLO11 instance segmentation module to remove potential object features from the tracking process while maintaining computational efficiency. In this work, the class “Person” is considered a dynamic object due to its predominance in both the evaluated TUM RGB-D sequences and the real-world robotic experiments.

The proposed system supports both RGB-D and stereo camera configurations and is evaluated on challenging dynamic sequences of the TUM RGB-D dataset and on a real mobile robot equipped with a stereo camera, demonstrating robustness and applicability in practical scenarios.

The contributions of this work are as follows:

- Dynamic object removal using YOLO11 instance segmentation, improving the performance of ORB-SLAM3 while preserving near-real-time tracking performance.
- Support for RGB-D and stereo cameras.
- Evaluation using TUM-RGB-D public datasets to validate improved localization accuracy and efficiency over ORB-SLAM3 and other semantic methods.
- Experiments with a robotic platform to validate improvement in localization using a stereo camera.
- The proposed solution and the ROS2 node are available on GitHub [9,10], respectively.

This work is divided as follows. After a brief introduction, Section 2 shows recent works related to the improvement of SLAM algorithms using deep learning techniques. Section 3 presents the architecture of the system developed in this work. Section 4 presents the results of the experiments and Section 5 analyzes and compares the results with other recent works. Finally, Section 6 concludes this work and presents ideas for future development.

## 2. Related Works

### 2.1. YOLO-Based SLAM for Dynamic Environments

Non-static objects affect feature-based V-SLAM systems as most of these algorithms assume that the features in the scene are static and can be used to estimate the robot’s position. Deep learning can be used to address these challenges, as the semantic information of the environment can be used to identify the dynamic features and remove them from the pose and map estimation.

YOLO plays an important role in addressing the dynamic problem in SLAM, and many researchers use this framework integrated with ORB-SLAM to improve performance in these scenarios. For instance, Refs. [11,12,13,14,15] used YOLO5 detection and segmentation modules to remove dynamic features from the scene. Ref. [16] used YOLO7 detection to remove dynamic features and employs semantic information, optical flow, and depth data to filter dynamic regions. YOLO8 semantic segmentation is used in [17,18,19,20] to filter out dynamic features. YOLO9 segmentation is integrated into the ORB-SLAM2 framework in [21] to filter out dynamic features during tracking. Ref. [22] also used YOLO to separate static and dynamic features.

YOLO11 is being studied to improve SLAM performance. For instance, Ref. [6] integrated YOLO11-based detection into the ORB-SLAM2 framework and achieved improvements in dynamic environments using a public dataset; however, it is limited by the older ORB-SLAM version and does not support stereo cameras. Ref. [7] used YOLO11 on ORB-SLAM3, not to remove dynamic features, but to improve the performance in low-texture scenarios. A more recent work [8] used YOLO11 segmentation to address the dynamic limitation, but it does not work in real time and also supports only RGB-D cameras.

These limitations highlight the importance of studying a more general and efficient SLAM solution based on recent YOLO versions, such as YOLO11, while supporting more camera configurations, maintaining real-time performance, and demonstrating robustness in real-world robotic scenarios.

### 2.2. Other Deep Learning Techniques Integrated into SLAM

To address these challenges in dynamic environments, other deep learning techniques have been increasingly adopted, as semantic information provides detailed insights into the objects observed by the robot and their spatial relationship to the camera.

RDS-SLAM [23] introduces semantic and optimization threads to remove dynamic features, but at high computational cost and without stereo support. DynaSLAM [24] integrates Mask R-CNN into the ORB-SLAM2 framework to remove dynamic objects but does not achieve real-time capability. USD-SLAM [25] adopts the SegGPT segmentation model to exclude moving features, while CS-SLAM [26] uses the Cross-SegNet network and an auxiliary mask to filter out dynamic elements, yet both approaches fail in real-time performance.

Lightweight approaches have also been explored. SE2-LET-VINS [27] combines SSD detection with geometric and IMU data, achieving good results in monocular setups but lacking support for other camera types. Ref. [28] addd object detection threads assisted by IMU, yet faces low detection accuracy and no instance segmentation. DOA-SLAM [29] integrates FastInt-based segmentation with static feature tracking, though at high computational cost. More recent efforts [30] adopted efficient architectures, such as Ghost Convolution and attention modules, to reduce complexity while preserving accuracy.

Other recent works integrated the Segment Anything Model (SAM) into ORB-SLAM to address dynamic environments. Zheng et al. proposed a framework that combines optical-flow-based motion estimation with coarse-to-fine SAM segmentation to detect and remove dynamic regions [31]. However, the code is not publicly available, and the computational time analysis is not clearly reported.

DZ-SLAM integrates fast SAM-based segmentation with optical flow to enhance ORB-SLAM3 in dynamic scenes [32], but the associated computational cost prevents real-time performance. In the same way, DN-SLAM combines SAM-driven semantic segmentation with optical flow refinement and implicit scene modeling to more precisely remove dynamic features and generate dense maps; however, it also does not support real-time performance [33].

Table 1 summarizes recent deep-learning-based SLAM approaches for dynamic environments. All of these methods demonstrate the potential of deep learning and semantic information to enhance ORB-SLAM in dynamic environments, although most compromise real-time operation due to computational complexity, limited code availability, or lack of support for multiple camera configurations. In this context, the present study proposes the use of YOLO11 to improve the system performance while maintaining real-time feasibility.

## 3. Materials and Methods

### 3.1. System Architecture

**Figure 1:**
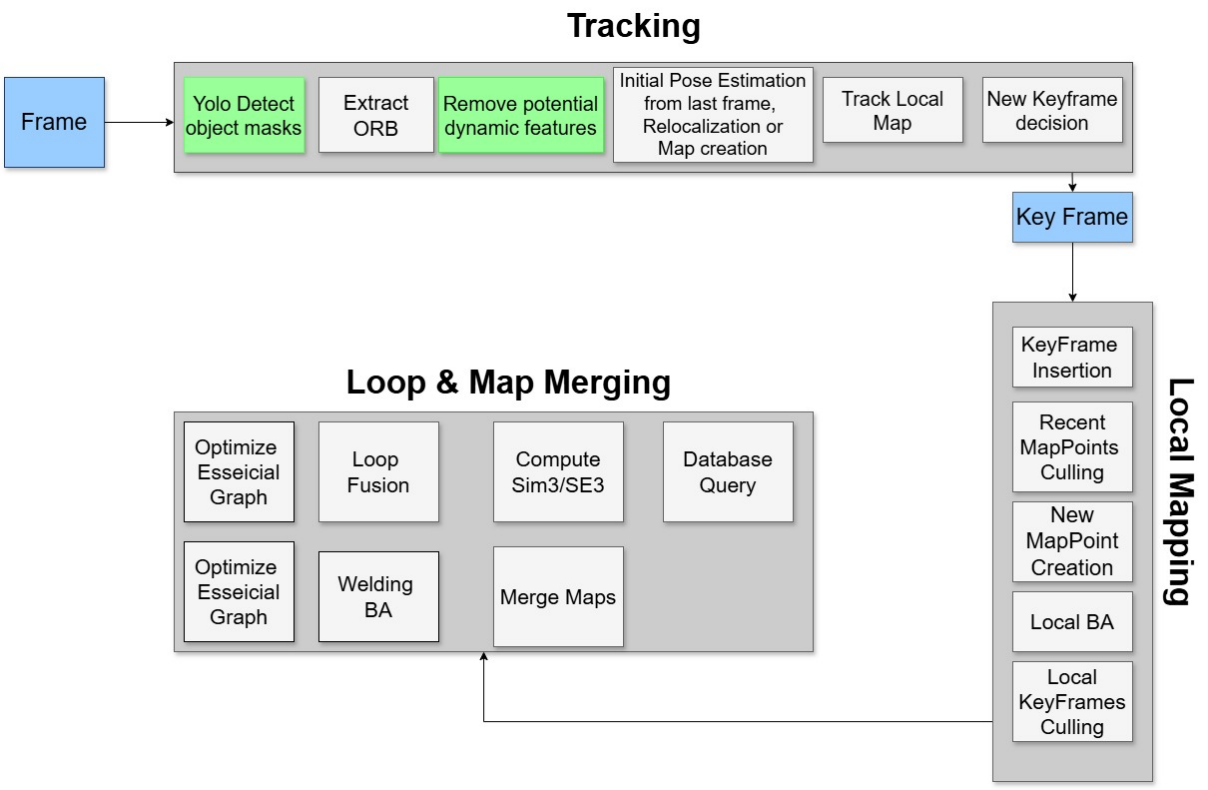
YOLO11-ORB-SLAM3 framework. The proposed YOLO detection and the object removal modules are marked by the green boxes.

YOLO11-ORB-SLAM3 integrates YOLO11 instance segmentation to identify and remove potential dynamic features. In the framework overview shown in Figure 1, the newly integrated modules are visually indicated by green boxes.

The modified tracking thread performs several tasks:

- Performs YOLO11 instance segmentation to remove potentially dynamic features.
- Determines whether the current frame should be selected as a keyframe.
- Estimates the pose of the current frame relative to the active map.
- Computes the frame’s velocity and acceleration.
- Attempts to relocalize the frame using all stored maps when the track is lost.

### 3.2. Instance Segmentation Module

YOLO11-ORB-SLAM3 considers “Person” as the dynamic class to be filtered, but this parameter is configurable in the implementation. This class was selected because it is the predominant dynamic class in both the evaluated TUM RGB-D sequences and the real-world robotic experiments, but the system is compatible with multi-class dynamic feature filtering. The YOLO11 lightest model, *yolo11n-seg.pt*, was used as the pretrained model due to its robust performance.

**Figure 2:**
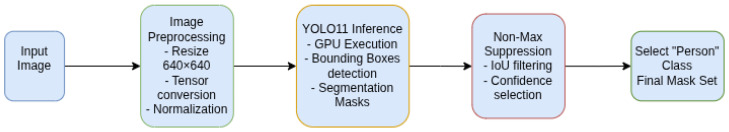
Overview of the YOLO11-based instance segmentation module added to the ORB-SLAM3 tracking process.

The diagram described in Figure 2 shows the process of getting the masks. The frame is first resized and normalized, then the frame is sent to the GPU processor where the YOLO11 inference is executed and the bounding boxes and segmentation masks are computed. These masks and bounding boxes are evaluated using Non-Maximum Suppression (NMS) to eliminate redundant detections by comparing their Intersection over Union (IoU) and keeping only the bounding boxes with the highest confidence score.

The integration of this module inside the ORB-SLAM3 tracking thread is described in Algorithms 1 and 2. For each frame, the instance segmentation masks are generated and saved in a vector containing all dynamic regions. In the tracking thread, ORB features detected inside these masked regions are filtered out before the pose estimation, so the algorithm only considers the static features.
**Algorithm 1** YOLO11-based dynamic region segmentation.**Require:** Current image $I_{t}$, YOLO11 model M, score threshold $t_{s}$, IoU threshold $t_{iou}$,      segmentation threshold $t_{seg}$**Ensure:** Set of dynamic masks D      **Image Preprocessing**  1: Resize $I_{t}$ to 640×640  2: Convert image to RGB format  3: Create input tensor *T*      **YOLO11 Inference**  4: Move tensor *T* to GPU  5: Perform inference to obtain detections and segmentation outputs      **Non-Maximum Suppression**  6: $F\leftarrow \mathrm{NMS}\text{\_}\mathrm{Seg}(O,{⊔}_{\int },{⊔}_{\rangle ≀⊓})$      **Dynamic Mask Generation**  7: Initialize dynamic mask set D←Ø  8: **for all** detections d∈Fdo  9:       Extract class ID *c* and confidence score  10:     **if** *c* corresponds to a dynamic object **then**  11:            Extract segmentation ROI  12:            Apply sigmoid activation to segmentation logits  13:            Binarize mask using threshold $t_{seg}$  14:            Resize mask to original image resolution  15:            Add mask to D  16:     **end if**  17: **end for**                **return**  D

**Algorithm 2** ORB-SLAM3 tracking with dynamic feature filtering.
**Require:** Current image $I_{t}$, dynamic masks D, SLAM map S**Ensure:** Updated camera pose and tracking state      **ORB Feature Extraction**  1: Extract ORB keypoints K from $I_{t}$      **Dynamic Feature Filtering**  2: **for all** keypoints k∈Kdo  3:       **if** *k* lies inside any mask in D **then**  4:            Remove *k* from K  5:     **end if**  6: **end for**      **Pose Estimation and Map Update**  7: Match remaining keypoints with map S  8: Estimate camera pose  9: Update tracking and mapping states


In the proposed architecture, only the features inside segmentation masks are removed, without applying spatial dilation or a margin tolerance. This design choice was motivated by the need to preserve a sufficient number of valid static features, particularly in low-texture environments. Although segmentation inaccuracies at object borders may occasionally leave residual features close to dynamic regions, the ORB-SLAM3 geometric consistency and temporal feature validation can mitigate this impact. Therefore, restricting the masking operation to the exact segmented regions provides a balanced trade-off between dynamic feature suppression and static feature preservation, ensuring stable tracking performance without introducing aggressive feature loss.

The Non-Maximum Suppression parameters used in the validation tests are listed in Table 2. These parameters were experimentally chosen considering the requirements of performance and accuracy.

All YOLO11 inference and ORB-SLAM3 processing were executed on a computer running Ubuntu 22.04 OS and an NVIDIA GeForce RTX 5060 GPU and 8 GB RAM. The YOLO11 instance segmentation model was executed on the GPU using TorchScript, while the ORB-SLAM3 tracking thread ran on the CPU.

The output masks are used in the tracking thread of ORB-SLAM3. The modified tracking thread works as follows:

- Performs the YOLO instance segmentation using the instance segmentation module described in Figure 2.
- Selects the dynamic objects and removes the features inside their related masks.
- Decides whether the current frame will be considered a keyframe and estimates its pose relative to the active map, as well as its velocity and acceleration.
- Verifies if tracking is lost and executes the relocalization process if needed. If the relocalization fails, this thread is responsible for saving the map in the Atlas and starting a new one.

**Figure 3:**
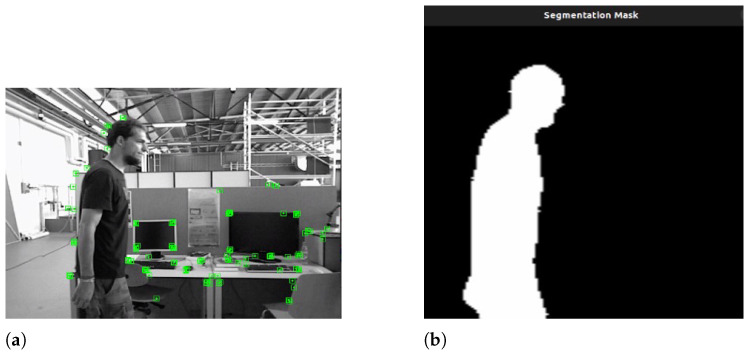
YOLO11-ORB-SLAM3 instance segmentation example. (**a**) Example of YOLO11-ORB-SLAM3 frame. The green points are the features detected by the algorithm. (**b**) Output of the segmentation module. This mask represents the person in the scene.

The output of the process is a frame free of potential non-static features. Figure 3a,b show an example of a semantic mask obtained by the semantic module and the filtering of dynamic objects from the ORB-SLAM3 frame.

### 3.3. TUM Dataset

TUM RGB-D dynamic scenes [34] were used to validate the performance of YOLO11-ORB-SLAM3 in RGB-D mode. TUM is a well-known resource that provides both RGB-D images and corresponding ground-truth data, designed to evaluate Visual SLAM techniques.

The sequences *f2-desk_with_person*, *f3-walking-xyz*, and *f3-walking-halfsphere* were considered to evaluate the proposed solution, as they are commonly used in Visual SLAM works and can be a reference to compare the performance of YOLO11-ORB-SLAM3 with other works. Each set consists of a sequence of 640 × 480 images recorded at a frame rate of 30 frames per second. The experiments using the TUM dataset were conducted by executing each sequence five times and selecting the median result for analysis.

**Figure 4:**
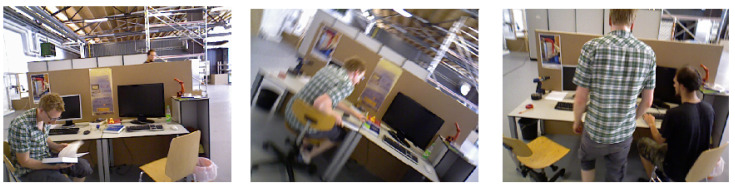
F3-walking-halfsphere sequence.

**Figure 5:**
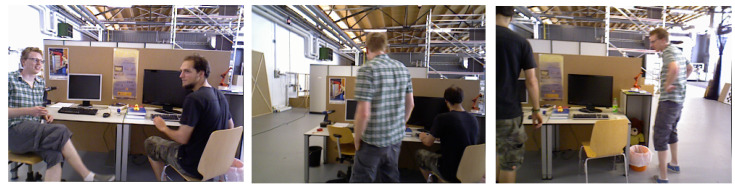
F3-walking-xyz sequence.

The *f3-walking* sequence represents highly dynamic scenarios involving two individuals walking in an office environment. In *f3-walking-halfsphere*, the camera remains in the same position, but rotates across the roll, pitch, and yaw axes. In *f3-walking-xyz*, the camera moves while people walk in and out of the place. Figure 4 and Figure 5 show a frame of *f3-walking-halfsphere* and *f3-walking-xyz*, respectively.

**Figure 6:**
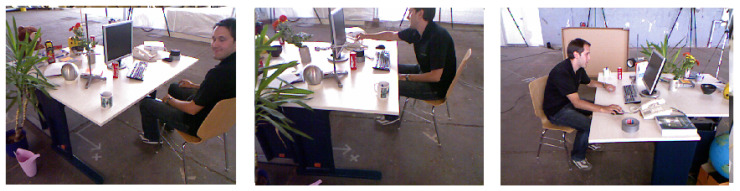
F2-desk_with_person sequence.

The *f2-desk_with_person* consists of a scene containing common office furniture, such as chairs, desks, and other objects, accompanied by a moving person. This sequence is represented in Figure 6.

For each sequence, the ORB-SLAM parameters were edited to improve the algorithm performance, but the same parameters were used in the proposed method and the original ORB-SLAM3 algorithm to maintain the comparison consistency. Table 3 shows the most important parameters used in each sequence. Notice that for F2-desk_with_person, the number of features, the initial threshold, and the minimum threshold of the FAST algorithm needed to be changed to address the overall low texture of this sequence. The complete parameters list can be found in the GitHub of this work.

### 3.4. Robotic Platform

**Figure 7:**
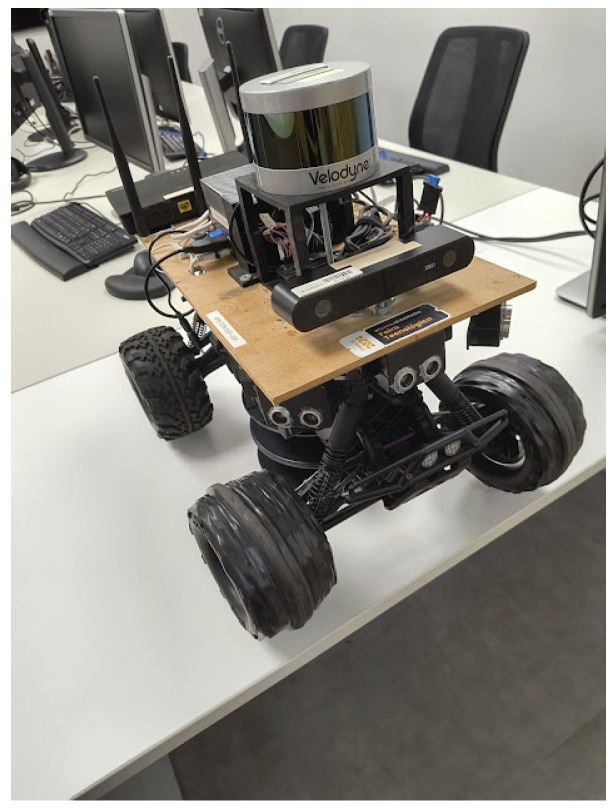
Real robot platform.

Figure 7 illustrates the robotic platform used to evaluate the algorithm. Built using the SAVAGE FLUX HP 1/8 chassis, it adopts the Ackermann steering geometry, and its sensor specifications are listed in Table 4.

The platform has four control modules. The Raspberry Pi 4 manages the LiDAR, both GPSs, and the IMU. The NVIDIA Jetson AGX Orin is dedicated to processing camera streams, operating the joystick, and saving data to external storage. The ESP32 is responsible for driver control and velocity of the robot. The STM32F401CC processes the ultrasonic sensors data. ROS2 is used to access all sensor information.

The system is integrated to a ROS2 humble node, which handles the image acquisition and synchronization. The experiments executed with the real robot involved recording an ROS2 bag containing the images and the reference odometry. The original algorithm and the proposed solution were executed five times, and the median of the results was considered for analysis.

**Figure 8:**
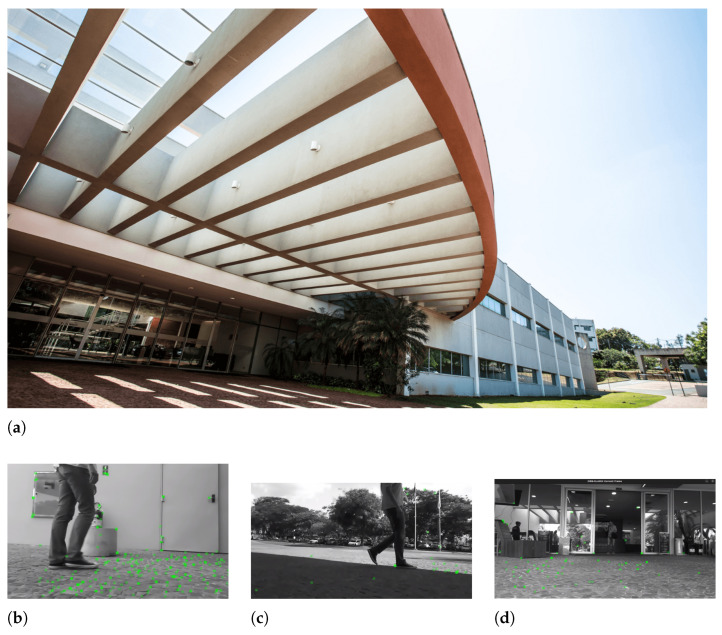
Images captured in a real-world scenario. (**a**) Recording area [35]. (**b**) People walking near a door. (**c**) People walking near a park. (**d**) People leaving through a door.

The test was conducted in a dynamic environment in which the robot moves near a parking area with people walking around and exiting through a door. Figure 8b–d, shows an example of the dynamic environment with persons walking in the scene. Figure 8a shows a view from the area where the images were taken.

The robot follows a circular path, moving at a constant speed of 0.5 m/s, passing through a parking area and the entrance of a building. For evaluation, the reference trajectory was taken from the stereo odometry estimated by the ZED framework. Notice that the ZED visual odometry does not represent absolute ground truth as it has its intrinsic error. Instead, it provides a consistent and repeatable reference for comparative evaluation as both ORB-SLAM3 and YOLO11-ORB-SLAM3 were evaluated against the same reference trajectory.

The stereo camera acquired images at a resolution of 1280 × 720 and at a frame rate of 10 FPS, with the reduced frame rate resulting from the high internal processing. An ROS2 bag, available on GitHub [9], was recorded to ensure the reproducibility of the experiment. Identical ORB-SLAM parameters were used for the baseline system and the YOLO11-ORB-SLAM3 variant.

Table 5 summarizes the camera parameters and the ORB-SLAM3 parameters used in the real robot evaluation. The camera parameters were defined according to the ZED2i camera model, while the ORB parameters were empirically selected for this environment, which presents good illumination conditions but an overall low-texture scene.

To compensate for the limited texture, both the initial FAST threshold and the minimum FAST threshold of the ORB extractor were reduced to allow more pixels to be identified as corners and improve the tracking performance. The complete list of parameters used are available in the GitHub.

## 4. Results

This section reports the results of experiments with YOLO11-ORB-SLAM3. Section 4.1 presents the evaluation results using the TUM dataset, including a comparative analysis with the original ORB-SLAM3. The results of the real robot platform experiments are discussed in Section 4.2.

### 4.1. TUM Datasets Experiments

#### 4.1.1. F3-walking-xyz Sequence

YOLO11-ORB-SLAM3 was tested in the F3-walking-xyz sequence and compared to the baseline ORB-SLAM3. Table 6 presents the absolute pose error (APE) and relative pose errors (RPEs). Both algorithms were tested under the same ORB parameter settings.

**Figure 9:**
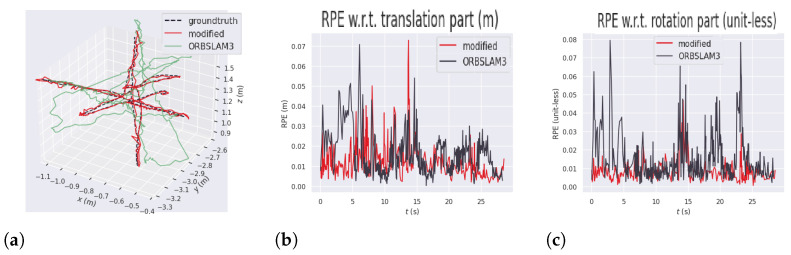
Results for absolute pose error (APE), relative pose error in translation, and relative pose error in rotation for the f3-walking-xyz sequence. (**a**) APE trajectory comparison in f3-walking-xyz. (**b**) Relative pose error (RPE) in translation for the f3-walking-xyz. (**c**) Relative pose error (RPE) in rotation for the f3-walking-xyz.

The absolute pose error (APE) measures the overall deviation between the estimated and ground-truth trajectories, whereas the relative pose error (RPE) captures the discrepancy between the estimated and ground-truth poses at each time step. APE provides a global assessment of trajectory accuracy, while RPE reflects the precision of individual pose estimates and the overall consistency of the trajectory. Figure 9 illustrates the estimated trajectory and the corresponding APE values for this dataset. In Figure 9b,c, the horizontal axis represents time in seconds, while the vertical axis corresponds to the error magnitude. The black line represents the errors of the original ORB-SLAM3 and the red line represents the errors of the modified version. In Figure 9a the x, y, and z axes correspond to the x, y, and z position of the robot in the map, respectively. The black dashed line represents the ground truth while the green and red lines represent the position tracked on original ORB-SLAM3 and the modified version, respectively.

The results showed a significant improvement in all the error metrics in the proposed method. This happens because this sequence presents the most dynamic scenario in which both the camera and the persons in the scene keep moving during the whole recording. Figure 9a shows the 3D representation of the trajectory measured by the original ORB-SLAM3, the YOLO11-ORB-SLAM3, and the reference trajectory. This figure highlights the difficulty of the original ORB-SLAM3 in keeping the trajectory close to the ground-truth. The relative pose errors (RPEs), demonstrated in Figure 9b,c, show the measured error between the reference and the algorithms in each step of the execution time. This figure also demonstrates that the errors were significantly lower in the experiments with the proposed method.

#### 4.1.2. F3-walking-halfsphere Sequence

Experiments were executed in the F3-walking-halfsphere sequence using YOLO11-ORB-SLAM3 and the original algorithm. Table 7 shows the comparison of the error metrics between the experiments with the original ORB-SLAM3 and the method exposed in this work. Both algorithms were tested with the same ORB parameter settings.

**Figure 10:**
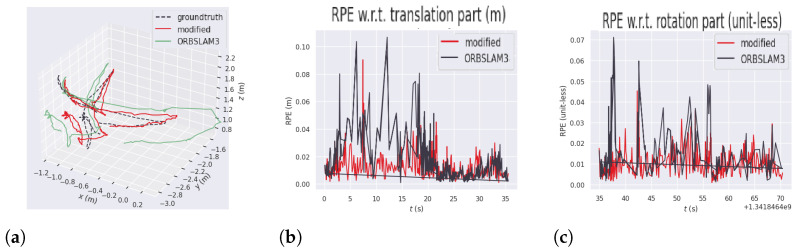
Absolute and relative pose errors results in the f3-walking-halfsphere dataset. (**a**) Absolute pose error comparison in f3-walking-halfsphere. (**b**) Relative pose error in translation in f3-walking-halfsphere. (**c**) Relative pose error in rotation in f3-walking-halfsphere.

Figure 10a shows that, compared to the original ORB-SLAM3, YOLO11-ORB-SLAM3 could follow the ground-truth 3D trajectory with better precision. In this figure, the green line represents the trajectory measured by ORB-SLAM3, while the red line and the dashed line represents the YOLO11-ORB-SLAM3 and the ground-truth trajectories, respectively. The proposed approach achieved a notable improvement in accuracy, with a reduction of 45.8% in APE compared to the baseline. This sequence is highly dynamic, so the original algorithm has more difficulty following the trajectory close to the reference and recovering from tracking loss. The part of the sequence in which the original ORB-SLAM3 loses the trajectory is also evident in this figure because this trajectory does not have a closed loop.

Figure 10b,c shows the relative pose error comparison between YOLO11-ORB-SLAM3 and the original algorithm in each step of the execution time. The error peaks in the original ORB-SLAM3 experiment, represented by the black lines, show the moments when the algorithm lost track. The error peaks in YOLO11-ORB-SLAM3, represented by the red lines, occur at moments of higher rotational motion, but in this case, the algorithm did not lose track, so the peaks are significantly lower than those of the original algorithm. Furthermore, the horizontal line observed in the ORB-SLAM3 relative pose error in translation and rotation plots indicates the point at which the algorithm lost tracking. At this moment, the keyframe timestamps become misaligned, resulting in the appearance of this line in the graph.

#### 4.1.3. F2-desk_with_person Sequence

The F2-desk_with_person sequence was tested using the original ORB-SLAM3 and the method proposed in this article. Table 8 shows the comparison of the APE and RPE results in the experiments using this sequence. Again, the same configuration was used in both tests.

**Figure 11:**
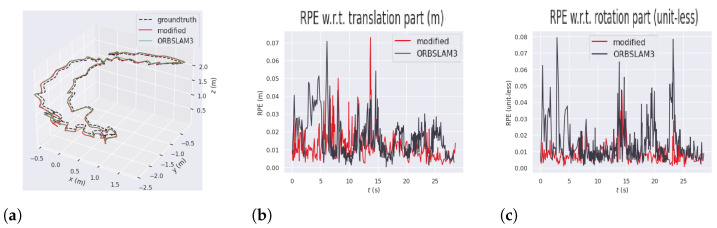
APE and RPE results on the f2-desk dataset. (**a**) Absolute pose error result in f2-desk. (**b**) Relative pose error for translation in f2-desk. (**c**) Relative pose error for rotation in f2-desk.

In this sequence, YOLO11-ORB-SLAM3 achieved better mean and median tracking errors; however, RMSE was 9.6% higher in the RPE translation component and 69.3% higher in the rotation component compared to the original algorithm. This occurs because objects considered as static are manipulated by the person in the scene and are not initially treated as dynamic. Moreover, the person occludes objects in the scene, which in some cases reduces the number of available features. This is reflected in the maximum rotation and translation errors, represented by the red line, shown in Figure 11b,c. In this segment, the camera moves behind the person, who shifts objects on the desk from one side to another, resulting in fewer image matches due to object motion and occlusion. Despite this limitation, YOLO11-ORB-SLAM3 still performed better in absolute pose errors, indicating robustness under challenging conditions.

#### 4.1.4. Tracking Time Comparison

The tracking time analysis presented in Table 9 indicates that, despite an increase in processing time, the system was able to perform in real time in most scenarios, using a 30 FPS camera as target. F3-walking-xyz sequence had the highest tracking time due to its dynamic nature, requiring constant instance segmentation and object removal. In contrast, the F2-desk sequence achieved the best computational time, highlighting the robustness of YOLO11, particularly given the presence of multiple objects in the scene.

### 4.2. Real Robot Platform

**Figure 12:**
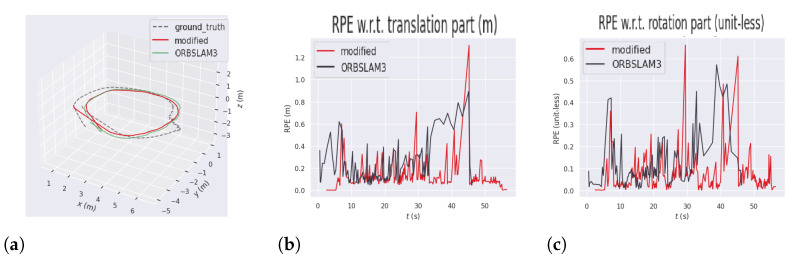
Absolute and relative pose errors comparison for the real robot experiment. (**a**) APE Trajectory comparison. (**b**) RPE Translation comparison. (**c**) RPE Rotation comparison.

The test was conducted by recording the images captured by the robot, using ROS 2 for data acquisition. Subsequently, experiments were carried out using the ORB-SLAM3 and YOLO11-ORB-SLAM3 algorithms. Table 10 shows the absolute and relative pose errors obtained in this test. Figure 12 represents the comparison of the 3D trajectory obtained during the experiments using ORB-SLAM3 and YOLO11-ORB-SLAM3. The ZED odometry reference is represented by the dashed line, while YOLO11-ORB-SLAM3 and ORB-SLAM3 are represented by the red and green lines, respectively. Figure 12b shows the RPE related to the translation part, measured in each step of the execution time, and Figure 12c describes the RPE related to the rotation part, measured in each step of the execution time. Table 11 shows the tracking time analysis of YOLO11-ORB-SLAM3 and the original algorithm experiments.

The results indicate a 33.3% improvement in the rotation RPE error and a 37.6% improvement in the translational error for the RMSE metric. However, the overall absolute pose error was 4.8% higher compared to ORB-SLAM3.

This discrepancy arises because the removal of dynamic objects leads to a low-texture environment, which degrades feature matching, and the robot loses tracking. These tracking failures are evident in the peaks of RPE translation and rotation errors, leading to a high RMSE and mean trajectory errors.

From a SLAM perspective, this behavior indicates a degradation in global map consistency rather than local motion estimation. While YOLO11-ORB-SLAM3 improves short-term pose estimation by suppressing dynamic features, the removal of image regions can reduce the number of features. In consequence, the system becomes more sensitive to tracking losses, which is propagated into global drift and higher absolute pose error (APE). Potential strategies to mitigate this limitation include improving the dynamic feature classification to only remove the features of the objects that are actually moving, or adaptive feature thresholds conditioned on texture availability. These strategies could help to preserve global consistency while maintaining robustness to dynamic objects, and will be explored in future work.

In both YOLO11-ORB-SLAM3 and ORB-SLAM3, the robot lost tracking, but in all experiments the algorithms were able to perform loop closure and recovering tracking. In the ORB-SLAM3 experiments, tracking was lost early in the sequence due to low-texture surroundings and abrupt motion, and again near the end when the robot passed a mirrored door and executed a sharp turn. This is reflected in the elevated initial RPE values. YOLO11-ORB-SLAM3 had more difficulty during sharp turns and in regions where objects were removed, as these areas had insufficient texture.

The influence of dynamic features on ORB-SLAM3 is evident in the peaks in the relative pose errors, corresponding to moments when people moved alongside the robot. The improved RPE performance of YOLO11-ORB-SLAM3 reflects improved local estimation of both position and orientation, as RPE evaluates the pose accuracy at each time step. However, the presence of error peaks negatively impacts the overall accuracy of the trajectory.

It is also important to note that the reported errors were calculated using the inertial visual odometry of the ZED framework, which may introduce its own inaccuracies. For this reason, it cannot be treated as the ground-truth, but it provides a reference for comparative evaluation. Moreover, the analysis emphasizes relative pose error (RPE), which focuses on short-term motion consistency and is less sensitive to global drift or accumulated reference inaccuracies. Establishing a ground-truth trajectory through precise measurement of the robot’s path would reduce uncertainty and allow a more rigorous evaluation of algorithm performance.

Table 11 indicates that the modified system exhibits a higher tracking time; however, in the considered robotic scenario, the camera operates at a low frame rate and the robot moves at a constant speed of 0.5 m/s, making an average processing time of 56.3 ms (approximately 17 FPS) sufficient to maintain stable tracking. In this context, real-time performance is defined as the ability to process each incoming frame without backlog or loss of synchronization with the sensor stream. This increase in processing time is expected due to the integration of a segmentation module designed to filter out dynamic features, thereby enhancing robustness in dynamic environments. It is acknowledged that lighter segmentation backbones could further reduce latency, at the cost of reduced segmentation accuracy, highlighting an inherent trade-off between robustness to dynamic objects and computational efficiency.

## 5. Discussion

With the evolution of SLAM techniques, interest in dynamic environments has increased, particularly due to their relevance in robotic applications such as autonomous driving. This section compares the performance of YOLO11-ORB-SLAM3 with other recent approaches.

The f3-walking-xyz APE error was used to compare the performance of the algorithms, as this dataset metric is present in most works in this area and provides a good indication of the performance in a dynamic, indoor, real-world scenario. Table 12 shows the results of recent works. As most of these algorithms are not open-source, the direct comparison with the same hardware could not be performed. For transparency, the hardware specifications reported in each work are explicitly listed in the table.

DYOLOv8-SLAM incorporates YOLO8 instance segmentation and a multi-view geometry to handle the non-static features in the frame. This method had the best performance on the f3-walking-xyz compared to the other methods; however, it does not have a tracking time analysis.

CS-SLAM [26] integrates Cross-SegNet to semantically segment and discard dynamic elements, using inter-frame mask comparison to improve robustness. Although it reports high accuracy, the lack of open-source availability and absence of tracking time metrics limit reproducibility and performance assessment.

SEG-SLAM [15] integrates YOLO5 into a fusion module for target detection and semantic segmentation to identify both explicit and potential dynamic objects. Although it performs well in the absolute pose error evaluation, the system lacks real-time capability.

DynaSLAM [24] is built upon ORB-SLAM2 and adds dynamic object detection via Mask R-CNN, resulting in a strong accuracy performance. However, the system was unsuitable for real-time applications. YOLOv9S [21] also uses ORB-SLAM2 and a geometric method to improve the dynamic object detection, and despite being able to run in real time for some sequences, the system only supports RGB-D cameras.

DFT-VSLAM [20] uses YOLO8 object detection task in combination with an optical flow mask to identify and remove dynamic features. The results demonstrate performance comparable to that of the proposed method, but it lacks a clear time consumption analysis. On the other hand, Ref. [18] uses the YOLO8 segmentation module and a contour-aware method to create a mask of the dynamic objects, but it does not provide a tracking time analysis.

DS-SLAM [36] incorporates SegNet-based semantic segmentation to remove prominent moving objects from the scene. However, it exhibits a higher error compared to YOLO11-ORB-SLAM3 and does not support real-time operation.

YOLO11-ORB-SLAM3 also outperforms USD-SLAM [12,14,25]. USD-SLAM utilizes the SegGPT segmentation model to remove dynamic regions from tracking, while [12,14] added a YOLO5-based module to filter out moving objects.

The goal of this comparison is not to establish a strict performance ranking, but rather to position the proposed YOLO11-ORB-SLAM3 within the current state of the art and highlight the trade-off between accuracy, computational cost, and real-time feasibility.

These comparisons demonstrate that most of the methods are still limited to RGB-D camera support and are still time-consuming. Moreover, the proposed approach aligns with current research trends and achieves favorable tracking time performance.

### Limitations

Although the proposed YOLO11-ORB-SLAM3 framework demonstrates improved robustness in dynamic environments while preserving near real-time performance, some limitations remain.

The current implementation focuses on filtering a single dynamic class, namely *Person*. This choice was motivated by the predominance of human motion in both the TUM RGB-D sequences and the real-world robotic experiments considered in this work. Although the system is compatible with multi-class dynamic filtering, additional experiments can be executed to evaluate its performance under multi-class scenarios. In particular, the impact of multiple dynamic object categories on tracking accuracy, computational cost, and real-time feasibility remains an open issue that should be investigated in future work.

The instance segmentation masks are applied without boundary refinement, which can introduce inaccuracies. Although the geometric consistency checks and temporal validation mechanisms of ORB-SLAM3 help mitigate this effect, more advanced mask refinement strategies could further improve robustness in highly cluttered or crowded scenes. In the same way, the current implementation does not verify whether the objects are actually moving, which could improve the accuracy in cases where there are “static” objects moving and “dynamic” object stationary. Motion validation mechanisms, such as optical flow, can be studied to fill this gap.

In addition, the ZED2i odometry used in the proposed system can introduce errors, so the reported error metrics should be interpreted as relative performance indicators rather than absolute ground-truth comparisons. Future evaluations could benefit from more accurate reference systems, such as motion capture setups, RTK-GPS, or high-fidelity simulation environments.

Although the proposed approach was validated on both public datasets and a real robotic platform, the evaluation did not include a quantitative comparison with large foundation models and other YOLO versions. Nevertheless, since the complete implementation and experimental pipeline are publicly available, future works can readily extend this study by conducting comparative evaluations with other YOLO versions or foundation models under identical conditions.

Finally, from the experimental analysis, it is also possible to identify that the method performs well in scenarios where dynamic objects occupy a small portion of the scene and where sufficient static background features remain visible for tracking, which is compatible with indoor scenarios. However, as the proportion of dynamic features increases or when large portions of the image are continuously occluded, the tracking accuracy decreases and a temporary loss of accuracy can occur.

## 6. Conclusions

YOLO11-ORB-SLAM3 is an efficient method to handle dynamic scenarios in V-SLAM. The results show that the proposed method achieved the goal of enhancing the performance of ORB-SLAM3 through dynamic object filtering using semantic segmentation.

Validation of YOLO11-ORB-SLAM3 in public RGB-D image datasets indicates significant improvements in pose estimation accuracy compared to ORB-SLAM3 and recent work. Furthermore, YOLO11-ORB-SLAM3 maintained computational efficiency, enabling real-time operation across multiple scenarios. Experiments with YOLO11-ORB-SLAM3 in the TUM dataset could achieve an absolute pose error 93.6% lower than ORB-SLAM3 in the f3-walking-xyz sequence. F2-desk sequence had the best tracking time, performing at a rate of 33.51 FPS. This result highlights the good performance of YOLO11, as this sequence contains more objects that are not considered dynamic in the scene than the others.

The extension of the system to stereo cameras and the validation of this solution in a real robot platform was also achieved. Validation with the real robotic platform shows that YOLO11-ORB-SLAM3 improved the relative pose error in 53.9% compared to the original algorithm. The APE was slightly higher for the modified approach due to the short distance and time of the trajectory and the reduction in the overall scene texture caused by dynamic object masks. Nonetheless, the system successfully operated in real time despite this constraint.

Future developments include integrating IMU data to improve robustness under low-texture conditions and a quantitative comparison with other YOLO variants. Optical flow techniques can also be employed to detect moving objects and remove them from the ORB-SLAM3 tracking process. In addition, the semantic information can be utilized to generate a comprehensive semantic map that describes the location and geometry of static objects.

## Figures and Tables

**Table 1:** Comparison of recent deep-learning-based SLAM methods for dynamic environments.

Method	Backbone	Camera Type	Dynamic Handling	Real-Time	Code
DynaSLAM [24]	Mask R-CNN	RGB-D	Instance Segmentation	No	Yes
RDS-SLAM [23]	CNN	Monocular	Semantic Removal	No	No
USD-SLAM [25]	SegGPT	RGB-D	Semantic Masking	No	No
CS-SLAM [26]	Cross-SegNet	RGB-D	Semantic Masking	No	No
YOLOv5-SLAM [11]	YOLOv5	RGB-D	Detection/Segmentation	Yes	No
YOLOv8-SLAM [17]	YOLOv8	RGB-D	Segmentation	Yes	No
YOLO11-SLAM [8]	YOLO11	RGB-D	Segmentation	No	No
DZ-SLAM [32]	SAM	RGB-D	Optical Flow + SAM	No	No
DN-SLAM [33]	SAM + NeRF	RGB-D	Dense Semantic SLAM	No	No
**Ours**	**YOLO11**	**RGB-D + Stereo**	**Instance Segmentation**	**Yes**	**Yes**

**Table 2:** YOLO11 instance segmentation parameters.

Parameter	Value
Intersection over Union	0.6
Segmentation threshold	0.5
Confidence score	0.5

**Table 3:** ORB-SLAM3 parameters used in the experiments.

Sequence	nFeatures	iniThFAST	minThFAST
F3-walking-xyz	1500	25	10
F3-walking-halfsphere	1500	25	10
F2-desk_with_person	2000	15	7

**Table 4:** Robotic platform sensor list.

Sensor	Model
GPS (ADR)	u-blox EVK-M8BL
GPS (Standard)	u-blox NEO-M8N
IMU	SparkFun Razor 9DOF
Joystick	DualShock 4
LiDAR	Velodyne VLP-16
Stereo Camera	Stereolabs ZED2i
Thermal Camera	FLIR ONE G3
Ultrasonic Sensor	HC-SR04
Velocity Sensor	F249

**Table 5:** Stereo camera and ORB-SLAM3 parameters used in the experiments.

Parameter	Value
Image resolution	1280 × 720
Focal length ($f_{x}=f_{y}$)	958.22
Principal point ($c_{x},c_{y}$)	(640.91, 350.25)
Stereo baseline (bf)	115.0
Number of ORB features	2500
Initial FAST threshold	15
Minimum FAST threshold	10

**Table 6:** f3-walking-xyz performance.

Error	Metric	YOLO11 ORB-SLAM3	ORB SLAM3	Improvement (%)
**APE** **(m)**	rmse	0.0174	0.2730	**93.6**
	mean	0.0145	0.2415	**94.0**
	median	0.0137	0.2256	**93.9**
**RPE Trans.** **(m)**	rmse	0.0154	0.0168	**8.3**
	mean	0.0128	0.0139	**7.9**
	median	0.0108	0.0112	**3.6**
**RPE Rot.** **(unit-less)**	rmse	0.0108	0.0191	**43.5**
	mean	0.0089	0.0144	**38.2**
	median	0.0076	0.0104	**26.9**

**Table 7:** f3-walking-halfsphere performance.

Error	Metric	YOLO11 ORB-SLAM3	ORB SLAM3	Improvement (%)
**APE** **(m)**	rmse	0.1355	0.2503	**45.8**
	mean	0.0962	0.2367	**59.3**
	median	0.0892	0.1990	**55.2**
**RPE Trans.** **(m)**	rmse	0.0159	0.0245	**35.1**
	mean	0.0127	0.0162	**21.6**
	median	0.0100	0.0107	**6.5**
**RPE Rot.** **(unit-less)**	rmse	0.0120	0.0193	**37.8**
	mean	0.0102	0.0143	**28.7**
	median	0.0090	0.0104	**13.5**

**Table 8:** f2-desk performance.

Error	Metric	YOLO11 ORB-SLAM3	ORB SLAM3	Improvement (%)
**APE** **(m)**	rmse	0.0069	0.0139	**50.3**
	mean	0.0049	0.0129	**62.0**
	median	0.0042	0.0122	**65.6**
**RPE Trans.** **(m)**	rmse	0.0073	0.0066	−***9.6***
	mean	0.0036	0.0053	**32.1**
	median	0.0028	0.0043	**34.9**
**RPE Rot.** **(unit-less)**	rmse	0.0171	0.0101	−***33.9***
	mean	0.0073	0.0085	**14.1**
	median	0.0051	0.0069	**26.1**

**Table 9:** Tracking time comparison.

Dataset	Tracking Time (ms)	ORB SLAM3	YOLO11 ORB-SLAM3
f3-walking xyz	mean	24.35	39.55
	median	25.70	43.32
f3-walking halfsphere	mean	21.81	33.33
	median	25.74	36.64
f2-desk	mean	23.10	27.88
	median	22.87	29.83

**Table 10:** Errors comparison with ORB-SLAM3 (Stereo).

Error	Metric	YOLO11 ORB-SLAM3	ORB SLAM3	Improvement (%)
**RPE** **Rotation**	rmse	0.1056	0.1584	**34.9**
	mean	0.0662	0.1080	**38.7**
	median	0.0444	0.0706	**37.2**
**RPE Trans.** **(m)**	rmse	0.1848	0.2959	**37.2**
	mean	0.1211	0.2162	**44.0**
	median	0.0857	0.1317	**34.9**
**APE** **(m)**	rmse	0.4664	0.4449	−***4.6***
	mean	0.4278	0.4101	−***4.3***
	median	0.4602	0.3892	−***18.2***

**Table 11:** Comparison of the tracking time (Stereo Experiment).

Tracking Time (ms)	ORB-SLAM3	YOLO11 ORB-SLAM3
mean	40.3	56.3
median	39.5	51.6

**Table 12:** Comparison of the absolute pose error with recent works.

SLAM Algorithm	APE RMSE (m)	Segmentation Model	Tracking Time Analysis	Hardware Information
DYOLOv8-SLAM [17]	0.0120	YOLOv8	No tracking time analysis	Intel Core i5
				Radeon HD 7500M/7600M GPU
CS-SLAM [26]	0.0140	Cross-SegNet	No tracking time analysis	NVIDIA GeForce GTX 4090
				16 GB RAM
SEG-SLAM [15]	0.0141	YOLOv5	No real-time performance	Intel i5-6300HQ
				NVIDIA GTX 960M
				16 GB RAM
DynaSLAM [24]	0.0150	Mask R-CNN	No real-time performance	Not reported
YOLOv9S [21]	0.0152	YOLOv9	Real-time in some cases	Intel i5
				NVIDIA RTX 3060 Ti
CA-SLAM [18]	0.0154	YOLOv8-SEG	No tracking time analysis	MD1 Ryzen 5 5600 6-core 32 GB RAM
				NVIDIA GeForce RTX 3060 GPU
DFT-VSLAM [20]	0.0164	YOLOv8	No clear tracking time analysis	Intel Core i5-11500H
				16 GB RAM
**YOLO11-ORB-SLAM3 (Ours)**	**0.0174**	**YOLO11**	**Real-time**	Intel Core i5
				NVIDIA GeForce RTX 5060
				8 GB RAM
YOLOv5 + Clustering [14]	0.0174	YOLOv5	Real-time in some cases	Intel Core i7-11700
				NVIDIA RTX 3060
				32 GB RAM
DSSLAM [36]	0.0247	SegNet	No real-time performance	i7 CPU
				P4000 GPU
				32 GB RAM
USD-SLAM [25]	0.0350	SegGPT	No real-time performance	Not reported
ORB-SLAM + YOLOv5 [12]	0.0530	YOLOv5.7-0	No tracking time analysis	NVIDIAGeForce RTX2080Ti
				16 GB RAM

## Data Availability

The images of the real robot platform experiment are available on GitHub [9].

## References

[1] Gao X., Zhang T. (2021). Introduction to Visual SLAM: From Theory to Practice.

[2] Al-Tawil B., Hempel T., Abdelrahman A., Al-Hamadi A. (2024). A review of visual SLAM for robotics: Evolution, properties, and future applications. Front. Robot. AI.

[3] Campos C., Elvira R., Rodríguez J.J.G., Montiel J.M., Tardós J.D. (2021). Orb-slam3: An accurate open-source library for visual, visual–inertial, and multimap slam. IEEE Trans. Robot..

[4] Redmon J., Divvala S., Girshick R., Farhadi A. (2015). You Only Look Once: Unified, Real-Time Object Detection. arXiv.

[5] Khanam R., Hussain M. (2024). YOLOv11: An Overview of the Key Architectural Enhancements. arXiv.

[6] Zhou T., Wang C. Visual SLAM Enhancement in Dynamic Environments via YOLOv11 Feature Filtering. Proceedings of the 2025 4th International Conference on Intelligent Mechanical and Human–Computer Interaction Technology (IHCIT).

[7] Yao Y., Wang A., Yue X., Wang K., Li H., Wang Y. Adaptive-Optimized Semantic Point Cloud Mapping and Path Planning with YOLO11-seg. Proceedings of the 2025 International Conference on Advanced Mechatronic Systems (ICAMechS).

[8] Hu T., He Y., Liu F. Visual inertial SLAM algorithm with SuperPoint and semantic information. Proceedings of the 2025 International Conference on Sensor-Cloud and Edge Computing System (SCECS).

[9] Villegas R. (2025). ORBSLAM3_YOLO11. https://github.com/renatavillegas/ORBSLAM3_YOLO11.

[10] Villegas R. (2025). ORB_SLAM3_ROS2. https://github.com/renatavillegas/ORB_SLAM3_ROS2.

[11] Pan G., Cao S., Lv S. A Dynamic Visual SLAM System based on YOLOv5s and Scene Flow. Proceedings of the 2025 Joint International Conference on Automation-Intelligence-Safety (ICAIS) & International Symposium on Autonomous Systems (ISAS).

[12] Wang H., Du J. ORB-SLAM3 Dynamic Scene Reconstruction based on fused YOLOV5. Proceedings of the 2024 IEEE 7th Advanced Information Technology, Electronic and Automation Control Conference (IAEAC).

[13] Pan G., Cao S., Lv S., Yi Y. (2025). DEG-SLAM: A dynamic visual RGB-D SLAM based on object detection and geometric constraints for degenerate motion. Meas. Sci. Technol..

[14] Gan F., Xu S., Jiang L., Liu Y., Liu Q., Lan S. (2024). Robust visual SLAM algorithm based on target detection and clustering in dynamic scenarios. Front. Neurorobot..

[15] Cong P., Li J., Liu J., Xiao Y., Zhang X. (2024). SEG-SLAM: Dynamic Indoor RGB-D Visual SLAM Integrating Geometric and YOLOv5-Based Semantic Information. Sensors.

[16] Jie T., Gong G., Jie F., Shuang W. A Visual SLAM System in Dynamic Environments Based on ORB-SLAM3. Proceedings of the 2025 4th International Symposium on Computer Applications and Information Technology (ISCAIT).

[17] Kumar D., Muhammad N. (2023). Object detection in adverse weather for autonomous driving through data merging and YOLOv8. Sensors.

[18] Wisal M., Yan D., Yang D., Shah S.S., Mala B.A. (2025). CA-SLAM: Contour-Aware SLAM System Based on RGB-D Sensors in Dynamic Environment. IEEE Sens. J..

[19] Wang Y., Liu X., Zhao M., Xu X. (2024). VIS-SLAM: A Real-Time Dynamic SLAM Algorithm Based on the Fusion of Visual, Inertial, and Semantic Information. ISPRS Int. J. Geo-Inf..

[20] Cai D., Li S., Qi W., Ding K., Lu J., Liu G., Hu Z. (2024). DFT-VSLAM: A Dynamic Optical Flow Tracking VSLAM Method. J. Intell. Robot. Syst..

[21] Zhu Q., Zhao Y., Zhang H., Chen W. (2025). A dynamic SLAM algorithm based on improved YOLOv9S. Appl. Soft Comput..

[22] Li L., Zuo Z.K., Bu R.F., Zhang Y.J., Wang R.M., Tan E.L. (2025). An Illumination-Adaptive Visual SLAM Algorithm with Lightweight Object Detection for Complex Dynamic Environments. IEEE Trans. Instrum. Meas..

[23] Liu Y., Miura J. (2021). RDS-SLAM: Real-Time Dynamic SLAM Using Semantic Segmentation Methods. IEEE Access.

[24] Bescos B., Fácil J.M., Civera J., Neira J. (2018). DynaSLAM: Tracking, mapping, and inpainting in dynamic scenes. IEEE Robot. Autom. Lett..

[25] Wang J., Ren Y., Li Z., Xie X., Chen Z., Shen T., Liu H., Wang K. (2024). USD-SLAM: A Universal Visual SLAM Based on Large Segmentation Model in Dynamic Environments. IEEE Robot. Autom. Lett..

[26] Guo Z., Dong N., Zhang Z., Mai X., Li D. (2025). CS-SLAM: A Lightweight Semantic SLAM Method for Dynamic Scenarios. IEEE Trans. Cogn. Dev. Syst..

[27] Zhao W., Sun H., Ma S., Wang H. (2025). LET-SE2-VINS: A Hybrid Optical Flow Framework for Robust Visual–Inertial SLAM. Sensors.

[28] Wang S., Hu Q., Zhang X., Li W., Wang Y., Zheng E. (2025). LVID-SLAM: A Lightweight Visual-Inertial SLAM for Dynamic Scenes Based on Semantic Information. Sensors.

[29] Jia Z., Ma Y., Lai J., Wang Z. (2025). DOA-SLAM: An Efficient Stereo Visual SLAM System in Dynamic Environment. Int. J. Control Autom. Syst..

[30] Winata I.M.P.A., Oh J. (2024). Lightweight extraction and segmentation with ghost convolutional and attention module integration for visual SLAM. Int. J. Control. Autom. Syst..

[31] Zheng C., Zhang P., Li Y. (2025). Semantic SLAM system for mobile robots based on large visual model in complex environments. Sci. Rep..

[32] Zheng C., Zhang P., Li Y. (2024). DZ-SLAM: A SAM-based SLAM algorithm oriented to dynamic environments. Remote Sens..

[33] Zhang K., Huang K., Ruan C., Zang Q. (2024). DN-SLAM: A Visual SLAM with ORB Features and NeRF Mapping in Dynamic Environments. IEEE Sens. J..

[34] Sturm J., Engelhard N., Endres F., Burgard W., Cremers D. A benchmark for the evaluation of RGB-D SLAM systems. Proceedings of the 2012 IEEE/RSJ International Conference on Intelligent Robots and Systems.

[35] Instituto Eldorado (2025). Banner-Home. https://www.eldorado.org.br/en/.

[36] Yu C., Liu Z., Liu X.J., Xie F., Yang Y., Wei Q., Fei Q. DS-SLAM: A semantic visual SLAM towards dynamic environments. Proceedings of the 2018 IEEE/RSJ International Conference on Intelligent Robots and Systems (IROS).

